# Mechanisms of Protection Against Oxidative Stress During Hibernation

**DOI:** 10.3390/ijms27031319

**Published:** 2026-01-28

**Authors:** Irina Vladimirovna Shemarova, Elena Romanovna Nikitina

**Affiliations:** Sechenov Institute of Evolutionary Physiology and Biochemistry, Russian Academy of Sciences, St. Petersburg 194223, Russia; elena.nikitina@bk.ru

**Keywords:** hibernation, mitochondria, oxidative stress, antioxidant system

## Abstract

Hibernation—the ability of some animals to enter prolonged winter sleep—is a natural hypometabolic state that allows them to withstand adverse environmental factors (low temperatures, food and water shortages). The ability to hibernate is a consequence of adaptations accumulated over evolution at various physiological levels, among which molecular adaptation to hypoxia plays a key role, which eliminates not only the negative effect of oxygen deficiency on cells, but also the danger of oxidative stress (OS) after awakening. This aspect of hibernation is medically important because understanding the mechanisms underlying the adaptation of hibernating animals to hypoxia and OS can help address a number of important issues related to preventing post-hypoxic complications in people with chronic neurodegenerative and heart disease. The molecular basis of adaptation to hypoxia in hibernating animals is the presence of an effective antioxidant system (AOC) and regulatory mechanisms that provide extraordinary mitochondrial plasticity, which is especially pronounced when animals emerge from hibernation. This review summarizes and systematizes the latest advances in the study of mitochondria and antioxidant defenses during mammalian hibernation, primarily gophers—a common experimental model of hibernation.

## 1. Introduction

Hibernation is a state of slowed vital processes and metabolism in some animal species under the influence of adverse environmental factors—usually cold and food deprivation. Hibernation is characterized by a decrease in body temperature, slowed respiration and heart rate, and inhibited brain and muscular activity. It can last from several days to several months, depending on the animal species, external temperature, and other environmental conditions [1]. Deep hibernation is typical of rodents and insectivores. Some large mammals (bears, badgers, raccoon dogs) enter winter sleep, a form of hibernation with a lesser reduction in physiological processes and metabolism [2]. The sleep of these animals is less deep and lacks the distinct metabolic phases of small mammals such as rodents (ground squirrels) and insectivores such as hedgehogs and bats. Therefore, they are the experimental models of choice for studying hibernation in most laboratories. The biochemistry of metabolic processes during hibernation (torpor, torpid state) and between-bout awakenings in all these animals has been sufficiently well studied, allowing for the accumulation of experimental data from these subjects for subsequent extrapolation (taking into account data from molecular and genetic studies) to humans.

The hibernation model using experimental animals is of particular interest to clinicians, as the emergence from torpidity in hibernating animals (hibernators) functionally resembles the state of reperfusion in patients with ischemia–reperfusion syndrome, which is characterized by cellular processes associated with excessive ROS production. The fact that emergence from torpidity in hibernators is not accompanied by ROS overproduction is attracting increasing attention from geneticists and molecular biologists. Progress in understanding the existing differences between hibernating and non-hibernating animals and humans is associated with two important achievements: an understanding of the role of hibernators’ defense mechanisms against oxidative stress (OS) and the identification of the biochemical and molecular genetic features of their antioxidant system (AOS). The antioxidant system includes low-molecular (glutathione (GSH)) and enzymatic components (superoxide dismutase (SOD), catalase (CAT), glutathione peroxidase (GP), glutathione-S-transferase (GST), and glutathione reductase (GR)). The thioredoxin system and vitamins (A, C, and E) are also involved in the process of protecting tissues from reactive oxygen species (ROS) in hibernators. Despite intense interest in the regulation of ROS production in the mitochondria of hibernators, the role of antioxidant systems and their individual components in detoxifying excess ROS products during various periods of natural hibernation remains understudied.

Below, we summarize and analyze the literature on these conceptual aspects of hibernation.

## 2. The Role of Mitochondria and Mitochondrial Mechanisms in Protecting Hibernators from Oxidative Stress

**Figure 1:**
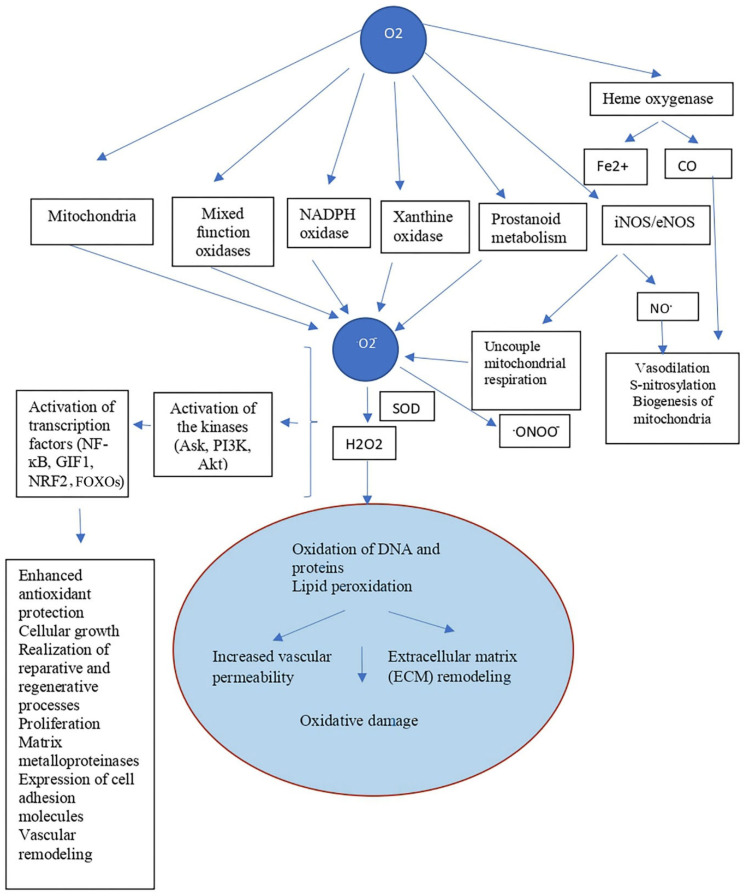
The dual role of O_2_ in mammalian cells: oxidative cell damage and ROS-induced cytoprotection. Intracellular utilization of O_2_ via mitochondrial monooxidase systems generates ROS, but O_2_ is also required for the production of two other gaseous transmitters: NO and CO. Some O_2_-produced reactive oxygen intermediates, such as H_2_O_2_, exert pluripotent effects on the cell: cytotoxic, for example, during oxidation of proteins, DNA and lipid peroxidation, or, conversely, adaptive, for example, by enhancing antioxidant defense. Ask1—apoptosis signal-regulating kinase 1 (ASK1); Fe^2+^—ferrous iron; HIF-1—hypoxia-inducible factor 1; Nrf2, NF-κB, FOXOs—transcription factors; iNOS/eNOS—inducible nitric oxide synthase/endothelial nitric oxide synthase; ONOO^−^—peroxynitrite anion; PI3K—phosphoinositide 3-kinase; CAT—catalase, SOD—superoxide dismutase; GP—glutathione peroxidase. The figure was partially constructed using the Servier Medical Art (https://smart.servier.com), licensed under CC BY 4.0 (https://creativecommons.org/licenses/by/4.0/ (accessed on 17 November 2025)).

Hibernating animals, under conditions that initiate natural hibernation (cold, food and water deprivation), initially enter torpor as the ambient temperature decreases—a state in which body temperature drops significantly, and metabolic and vital processes slow down [1,2]. To protect important biological molecules from degradation in response to starvation and cold stress, as well as to provide protection of cells from free radical processes (Figure 1), hibernators experience a decrease in mitochondrial activity. Under the influence of ROS (indirectly through modulation of tyrosine phosphatases and other regulatory proteins), the kinases Ask, PI3K, Akt and nuclear transcription factors (Nrf2, NF-κB, FOXOs) are activated, and the production of antioxidant defense enzymes (Figure 1), proteins of the HSP family and others cytoprotective molecules is enhanced [2,3,4].

Mitochondria, where cellular respiration and free radical oxidation processes occur, play a crucial role in the transition from wakefulness to hibernation in hibernators [5,6]. Since energy-dependent processes are reduced in hibernators, optimization of cellular metabolism is achieved through the accumulation of ATP in the mitochondrial matrix. Furthermore, mitochondria play a leading role in coordinating critical cellular functions, as they participate in intracellular signaling, which initiate a cascade of events aimed at protecting cells and transitioning to a new level of reduced metabolism, including enhancing antioxidant protection (Figure 1).

Mitochondria themselves, their metabolism and signaling functions have been studied quite well. However, the mechanisms of mitochondrial dysfunction during ischemia–reperfusion in non-hibernating animals and humans, as well as the mechanisms that ensure the extraordinary plasticity of mitochondria during hibernation, are only in the very early stages of study [7,8,9].

Previously, studies of mitochondria and the activity of oxidase systems revealed that during natural hibernation, the activity of NADPH oxidase and succinate oxidase decreases by approximately 2-fold [10]. However, the author of the study noted that cytochrome oxidase activity is much less dependent on hibernation [10]. It has now been established that the decrease in oxidative phosphorylation and the rate of ATP production in the mitochondria of hibernators occurs not due to a disruption of the barrier properties of the inner mitochondrial membrane, as occurs during hypoxia and ischemia–reperfusion [11], but due to the suppression of the activity of NADH dehydrogenase and succinate-ubiquinone oxidoreductase—key complexes of the electron transport chain (ETC), as well as sulfide:quinone oxidoreductase (SQOR), a sulfide-oxidizing enzyme that delivers electrons to the ETC with the participation of ubiquinone (coenzyme Q) [12].

**Figure 2:**
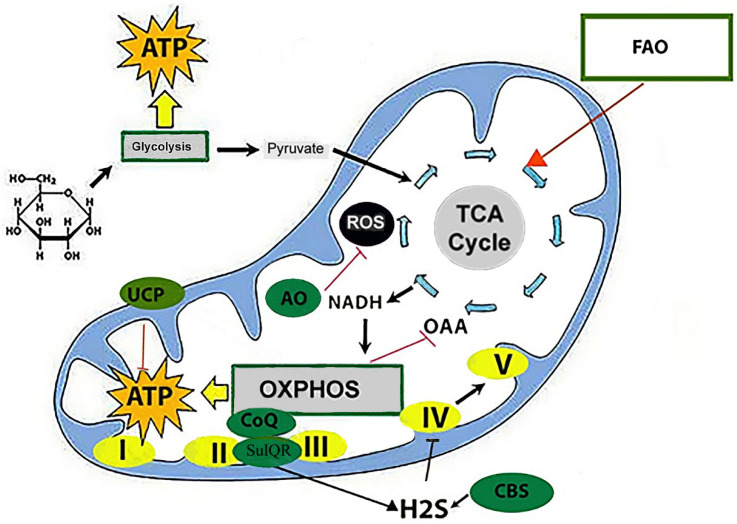
Mitochondrial respiration during hibernation. During the torpor phase, aerobic glycolysis is suspended, pyruvate production decreases, and fatty acids are involved in the formation of acetyl-CoA in the Krebs cycle. The predominance of anaerobic glycolysis and the decrease in pyruvate production lead to the inhibition of all stages of the tricarboxylic acid (TCA) cycle, including those that provide substrates to mitochondrial complexes I and II of the electron transport chain (ETC). The main regulator of the TCA is oxaloacetate (OAA); it recruits acetyl-CoA into the TCA and initiates the process. Under physiological conditions (normoxia), the cell maintains a balance between the formation of acetyl-CoA (from glucose, fatty acids, or amino acids) and the amount of OAA, an intermediate product of the TCA cycle. Elevated H2S levels, due to decreased efficiency of H2S oxidation by sulfide:quinone oxidoreductase SulQR and increased H2S production by cystathionine β-synthase (CBS), inhibit complex IV function. ROS production is reduced due to down-regulation of complex I, which normally produces a large proportion of ROS in the oxidative phosphorylation system (OXPHOS). The up-regulation of uncoupling protein (UCP) complexes in brown adipose tissue (BAT) adipocytes leads to uncoupling of OXPHOS, thereby altering its redox state and inhibiting ROS production. However, the uncoupling is not complete, but only reduces the transmembrane potential, preventing the reverse transfer of electrons inside complex I, the main source of ROS production by mitochondria. The β-oxidation of fatty acids (FAO) produces acetyl-CoA, which is included in the TCA (red arrow). The transfer of the acetyl residue from acetyl-CoA to oxaloacetic acid leads to the formation of citric acid. The energy for this reaction is supplied by the macroergic acetyl-CoA bond. ROS—reactive oxygen species; AO—antioxidants; CoQ—coenzyme Q; CBS—cystathionine β-synthase; NADH—nicotinamide adenine dinucleotide (in reduced form); FAO—fatty acid oxidation; H2S—hydrogen sulfide; OAA—oxaloacetate; OXPHOS—oxidative phosphorylation system; SulQR—sulfide:quinone oxidoreductase (SQOR, or SQR); UCP—uncoupling protein from UCP1 complexes in BAT or UCP3 in brain cells or skeletal muscles; I–V—respiratory complexes. The figure was partially constructed using the Servier Medical Art (https://smart.servier.com), licensed under CC BY 4.0 (https://creativecommons.org/licenses/by/4.0/ (accessed on 17 November 2025)).

The activity of the respiratory proteins of the oxidative phosphorylation system (OXPHOS) in mitochondria is regulated by the energy needs of the cells through retro-inhibition of oxaloacetate, which accumulates in the mitochondria due to a decrease in the activity of enzymes of the tricarboxylic acid (TCA) cycle, as well as through epigenetic regulation of the expression and post-translational modifications of intramitochondrial kinases and deacetylases [13]. In addition, a decrease in SQOR activity and an increase in the level of cystathionine β-synthase (CBS), an enzyme involved in the metabolism of sulfur-containing amino acids, leads to an increase in the level of hydrogen sulfide (H2S), which is a reversible inhibitor of cytochrome c oxidase at the end of the ETC [9]. Furthermore, up-regulation of uncoupling protein (UCP) complexes in brown adipose tissue (BAT) adipocytes leads to uncoupling of OXPHOS, thereby altering its redox state and inhibiting ROS production (Figure 2).

It has been noted that in hibernators, suppression of mitochondrial activity during torpor is the result of reduced catalytic activity of the ETC and TCA enzymes [8,9,14,15,16]. It is important to emphasize that both the suppression of mitochondrial function during torpor and the increase in their activity during awakening from hibernation occur in hibernators while maintaining sufficient energy reserves and without compromising the integrity of cellular structures caused by ROS, which are generated in the mitochondria during warming [8].

Another mechanism for protecting cells from ROS damage is the uncoupling of mitochondrial respiration, a process that ensures non-shivering thermogenesis and an increase in body temperature. The essence of this process is that most of the energy released during electron transfer in the mitochondrial respiratory chain is not spent on ATP synthesis, but is dissipated as heat. This process occurs in the mitochondria of specialized brown adipose tissue (BAT), which accumulates in large quantities during the summer-autumn period in the bodies of hibernating animals; it is present in newborns and young animals, as well as in mammals adapted to the cold [17]. A small amount of BAT is also found in human adults [18]. Uncoupling of respiration and phosphorylation in BAT is initiated by free fatty acids (FFA) and occurs with the participation of mitochondrial uncoupler proteins, which “spray” the proton gradient, transporting protons back into the mitochondria, bypassing ATP synthase [19,20,21]. In hibernators, the main natural uncoupler is uncoupling protein 1 (UCP-1), the activity of which increases with a decrease in body temperature [20]. It is known that the effect of cold in mice leads to an increase in ROS production, and thermogenesis is activated only under the condition of oxidative modification of UCP1 at the cysteine-253 [21]. It is noted that inactivation of ROS by the mitochondrial antioxidant MitoQ or restoration of the redox potential using N-acetylcysteine blocks this process in mice [21]. It is assumed that other uncoupling proteins (UCP2-5) operate in a similar way [22].

Uncoupling proteins are key regulators of energy metabolism and thermogenesis in hibernators. The increased regulation of UCP1-5 in various tissues during hibernation highlights their important role in adaptive heat production and metabolic control at low temperatures [23]. Moreover, by uncoupling oxidation and phosphorylation in mitochondria, uncoupling proteins, mainly UPC1, provide rapid warming of animals for out of torpor [24]. It is important to note that the period of awakening after torpor is accompanied by a marked increase in oxygen consumption necessary for maintaining thermogenesis by brown adipose tissue and skeletal muscles [25]. The development of negative consequences of reperfusion processes during this period is prevented by enzymatic antioxidant systems, including superoxide dismutase, catalase, and glutathione peroxidase [2].

In addition to antioxidants and uncouplers, telomerase also has the ability to reduce ROS levels, the mechanism of action of which is poorly studied, but it is known that the inhibitory role in OS is mainly played by the catalytic subunit of telomerase TERT [26]. In view of the above, it is important to note that activation of oxidative phosphorylation uncoupling, as a natural mechanism, can find application in medicine. Attempts are already being made to use various chemical agents to reduce ROS production in mitochondria in vivo. Thus, it has been established that the weak uncoupler 2,4-dinitrophenol (DNP) prolongs the lifespan of mice, reduces traumatic brain injury, and inhibits the development of a number of neurodegenerative diseases induced by OS. Under the influence of DNP, the levels of glucose, triglycerides, and insulin in blood plasma decrease. Unfortunately, it has been noted that DNP has a number of shortcomings that hinder its practical use [27]. Obviously, research in this area will continue.

## 3. Intracellular Antioxidant Systems in the Defense Strategy of Hibernators from Oxidative Stress

The antioxidant defense strategy of cells includes both balanced ROS formation and ROS detoxification mechanisms associated with the presence of an antioxidant system in cells [2,28]. The antioxidant systems (AOS) of hibernators include redox-active low-molecular-weight cellular compounds (glutathione (GSH), vitamins A, E, and C), as well as enzymatic systems for ROS metabolism (SOD, CAT, GP, etc.) [2,28,29].

ROS trigger many intracellular processes and activators of a number of proteins, of which nuclear factors AP-1, Nrf2, and FOXOs are key players in the regulation of stress-activated signaling pathways and the maintenance of cellular redox homeostasis [30,31]. These factors control the activity of genes responsible for cell survival and resistance to the effects of oxidative stress (Table 1). In response to a stress stimulus, they induce the expression of genes encoding antioxidant proteins such as catalase (CAT), mitochondrial manganese superoxide dismutase (MnSOD), glutathione-S-transferase (GST), NAD(P)H:quinone oxidoreductase 1 (NQO1), the negative regulator KEAP1, thioredoxin, small Maf proteins (MafF, MafK, and MafG), PA26 protein, and others (Table 1) [30,31].

It is important to note that many of the target gene products of nuclear factors activated by the OS have not yet been adequately studied, and their physiological role during hibernation is unclear. A number of them, such as the DEPP protein and metabolic proteins (Table 1), are activated by starvation under physiological conditions and may be associated with increased Gadd45a expression and activation of MAPKs [102]. Levels of other antioxidant stress proteins, such as SESN3, NQO1, CBR1, AKR1C1, AKR1B1, and GSTM1, depend on the metabolic status of cells and are associated with increased activity of AMP-activated protein kinase (AMPK) [39,103,104], which is important during hibernation [105]. Proteins that perform transport functions, such as MRP1, are also associated with antioxidant defense, and their induction can be used to improve the metabolic and antioxidant profile during ischemia–reperfusion injury [52].

Other proteins—the products of the target genes of nuclear factors activated by OS—play a multifaceted role in cells, and interest in them is primarily related to their possible involvement in cytoprotection in a number of neurodegenerative and other diseases associated with the development of OS [49]. Whether these proteins are involved in cytoprotection during hibernation is not yet known.

In hibernators, the transcription factors Nrf2 and FOXO3a, their target genes, and proteins expressed as a result of activation of these genes play a crucial role in protecting cells from oxidative stress (Table 1) [30,106].

In hibernators, the transcription factors Nrf2 and FOXO3a play a crucial role in protecting cells from oxidative stress [30,106]. It was found that during the late stage of torpor, there is a significant increase in the level of the small MafK protein, which leads to the activation of Nrf2 and its target, the catalase gene [30]. Furthermore, it was noted that torpor-awakening cycles in hibernators are accompanied by Nrf2-Keap1 protein–protein interactions and Nrf2 post-translational modifications, including serine phosphorylation and lysine acetylation, with the most intense reactions occurring during awakening, which corresponded to a sharp increase in oxygen consumption [30]. During the late phase of torpor, a 1.5-fold increase in the expression and phosphorylation of FOXO3a also occurs. Increased phosphorylation of FOXO3a at Thr-32 is accompanied by an increase in the expression of detoxification genes and a 1.2-fold increase in the phosphorylation of c-Jun mitogen-activated protein kinases, which alters the activity of many regulatory proteins that are located in the mitochondria or act in the nucleus [106].

Although many of the genes induced by FOXO3a encode enzymes directly involved in ROS detoxification (e.g., prdx-3, CAT, MnSOD), FOXO3a activation also initiates the expression of genes whose products negatively regulate the cell cycle (G0-to-G1 and G1-to-S transitions), including the cell cycle inhibitors p27 and p21. Moreover, in hibernators, FOXO3a activation can also promote DNA damage repair through the induction of Gadd45, a DNA-binding protein that is involved in the activation of DNA repair [106]. In addition, it has been shown that FOXOs proteins, through interaction with various coactivators, are involved in the regulation of energy metabolism and other cellular functions, including the regulation of apoptosis, modulation of lipid metabolism through interaction with the nuclear factor HNF4, and regulation of glucose levels through interaction with the Dyrk1 kinase and the SHP protein [107,108,109,110]. Although the role of FOXOs proteins in hibernation has been partially described, and their importance in antioxidant defense has been noted (since they provide mechanisms to mitigate the harmful effects of OS by modifying the expression and function of target genes), the involvement of these proteins in the regulation of lipid and carbohydrate metabolism requires study, since the metabolic shifts they initiate are crucial for maintaining the survival of animals during hibernation [106].

At the transcriptional level, the expression of antioxidant enzyme genes is regulated by the cis-active ARE (antioxidant response element) sequence [111]. In hibernators, the closest interaction is observed between genes whose promoter regions contain the ARE regulatory sequence and the transcription factor Nrf2 [112]. The result of this interaction is a decrease in the intensity of free-radical processes in mitochondria and the activation of antioxidant enzymes in cell membranes, which prevent lipid peroxidation in the early phase of torpor and inter-bout awakening [113]. NRF2 targets include genes encoding several antioxidant enzymes, such as NAD(P)H:quinone oxidoreductase-1 (NQO1), heme oxygenase-1 (HO-1), the cytoplasmic form of superoxide dismutase-1 (SOD-1), the catalytic and regulatory (Gclc, Gclm) subunits of glutamate cysteine ligase, a key enzyme in glutathione biosynthesis (Gclc, Gclm), as well as numerous genes associated with xenobiotic detoxification and the metabolism of chaperones, growth factors, receptor ligands, etc. (Table 1) [33,42,47,114,115,116]. It positively influences approximately 250 genes involved in maintaining antioxidant defense systems, participating in the detoxification of oxidants and xenobiotics, transport, cellular repair, etc. [116]. At the same time, NRF2 inhibits the expression of cyclooxygenase-2, iNOS, and the production of proinflammatory cytokines IL-1β, IL-6, and TNF-α, which also contributes to the cytoprotective function, including protection against oxidative stress [111,117,118]. Nrf1, which controls the activity of proteasomal protein degradation and transcription of globin genes, is also involved in the regulation of antioxidant enzyme production in non-hibernating animals [119]. It is important to note that the expression of genes encoding key antioxidant enzymes is under epigenetic control and depends on many other transcription factors regulated by changes in cellular redox homeostasis. Thus, an increase in SOD production in hibernators is associated not only with the activity of the transcription factor Nrf2, but also depends on post-translational regulation of its activity by sirtuins and the strengthening of intracellular signaling pathways associated with the transcription factors AP-1 and NF-κB [120].

Nrf2 activity depends on endogenous electrophilic compounds and, under normal physiological conditions, is suppressed by the specific repressor protein Keap1 (Kelch-like-ECH-associated protein 1), which is also a receptor for electrophilic compounds and an adapter protein for the ubiquitin ligase Cul3 [121]. ROS modify the sensitive thiol groups of Keap1 and thereby suppress its ability to inhibit Nrf2. This results in the release of Nrf2 from the cytoplasmic Keap1/Nrf2 complexes, its nuclear translocation and accumulation in the nuclear matrix, as well as heterodimeric reactions with regulatory ARE elements of target genes encoding protective proteins, including glutathione, and antioxidant enzymes (SOD, CAT, GP, GST, GR), as well as the disulfide-containing low molecular weight protein thioredoxin, which acts as an antioxidant [122,123]. It has been previously shown that the expression of Nrf2 protein in the gastrocnemius muscle of rats under ischemia–reperfusion conditions is significantly higher than under normal conditions, which indicates that the body can reduce the level of ROS by activating the Nrf2/Keap1 signaling pathway [124]. Hibernators also increase the expression of antioxidant enzymes in skeletal muscles, but whether this mechanism of antioxidant protection is associated with Nrf2/Keap1 is still unknown [125].

It is important to note that both FOXOs and Nrf2 are transcriptional activators of genes that, during the development of OS, perform functions beyond their best-known functions in the detoxification of electrophilic compounds and ROS. These include the regulation of metabolism by stimulating fat accumulation in BAT and the expression of lipid metabolism genes, which is of particular importance in preparing animals for hibernation [30].

Although increased expression of the Nrf2 and FOXOs transcription factor genes has been detected in hibernating mammals, the mechanisms underlying redox reactions and stress resistance in these mammals remain poorly understood [112].

During hibernation, Nrf-2 expression is known to increase in many tissues, including BAT, heart, liver, and skeletal muscle [125,126,127]. Heme oxygenase 1 (HO1) is a downstream target of Nrf-2. Increased HO1 synthesis, both at the transcriptional and elongation levels, has been observed in the brain and heart of hibernators [128]. Under physiological conditions, HO1 catalyzes the first stage of heme degradation, leading to the formation of antioxidants, and plays a crucial role in maintaining cellular redox homeostasis [129]. Recent studies have shown that in humans and mice, elevated HO1 mRNA levels are also associated with insulin resistance and obesity, physiological processes important for hibernators [130,131].

Elevated Nrf-2 levels were also detected in long-lived subterranean rodents, naked mole rats (*Heterocephalus glaber*). Although these animals do not hibernate, they are highly resistant to OS and, like hibernators, undergo metabolic depression in response to nutrient deprivation and decreased environmental temperature [132,133]. High Nrf-2 levels in mole rats correlated with decreased Keap1 expression and a multiple-fold increase in the expression of antioxidant proteins that are products of Nrf-2 downstream targets, including the genes hmox1, gsta1, and nqo1 [133,134]. It has been noted that in humans, a decrease in the level of the products of these genes—heme oxidase 1 (HMOX1), glutathione-S-transferase A1 (GSTA1), and NAD(P)H dehydrogenase 1 (NQO1), especially GSTA1—can lead to impaired liver function in detoxification processes and, possibly, a decrease in resistance to neoplasia [135]. Considering that a decrease in the expression of Nrf-2-controlled genes is characteristic of many pathologies in humans, including ischemia–reperfusion syndrome, naked mole rats can also become convenient objects (alternative comparative models) for studying the molecular mechanisms of cytoprotection during ischemia–reperfusion and other pathological conditions caused by hypoxia [136].

The question of the involvement of individual enzymatic components of the AOS in protection against OS during different periods of natural hibernation has also not been sufficiently studied. It is known that at certain stages of hibernation, the capabilities of the AOS are limited, which is associated with the suppression of metabolism and degradation of a number of enzymes and regulatory macromolecules. At the same time, numerous studies indicate an increase in the production of AOS enzymes and a decrease in ROS production during hibernation [137,138,139]. It has been noted that hibernating bats *Myotis lucifugus* exhibit a more than 50% decrease in the production of hydrogen peroxide (H2O2) in the brain, heart, and kidneys compared to similarly sized, but non-hibernating *Northern Short-tailed Shrews* [137]. The reduction in oxygen free radical production in bats is associated, in particular, with increased SOD content in the brain, heart, and kidneys, increased catalytic efficiency of the enzyme, and a decrease in free radical production per unit of oxygen consumed. Furthermore, tissues of hibernating bats exhibit lower levels of protein carbonylation in vitro, which may indicate increased resistance to OS [140].

In the erythrocytes of small ground squirrels (*Spermophilus pygmaeus*), the activity of CAT and SOD decreased during the awakening from hibernation, when the intensity of OS in the blood (Tb 25 °C) was maximum, after which the activity of antioxidants increased [141]. It is assumed that during hibernation, the level of antioxidant enzymes in hibernators is not high, in this case the primary role in protecting cellular structures from ROS reactivity is assigned to low-molecular-weight hydrophilic antioxidants [113].

It is important to note that increased antioxidant enzyme synthesis during hibernation and upon awakening is not observed throughout the body, but only in individual organs and tissues [142]. Thus, during hibernation, the content of CAT, SOD1, and SOD2 (MnSOD) in the BAT of ground squirrels increases by 1.5–2 times [34]. A tendency toward increased antioxidant enzyme levels was also observed in muscle tissue, but not in the liver or white adipose tissue [34]. This is likely due to the fact that basal SOD activity in the liver is approximately 4–6 times higher than in other tissues (kidneys, BAT, lungs, heart, and spleen), and this SOD activity may be sufficient to maintain antioxidant defense of the entire organism during hibernation [34]. However, although selective enhancement of AOS in a tissue-specific manner has been experimentally confirmed, it is also possible that alternative stress response mechanisms play a decisive role in maintaining the hibernation phenotype [34,139].

Thus, given the possibility of using hibernators as models for studying the mechanisms of protection against OS, which occurs during the development of many human pathological conditions, including ischemia–reperfusion syndrome, the study of AOS and the features of mitochondrial functioning during different periods of hibernation is of great medical and biological significance and will subsequently allow the use of the acquired knowledge in the strategy of post-hypoxic prevention and rehabilitation [7,143,144].

## 4. Prospects for Using Hibernators as Basis for the Development of New Therapeutic Strategies in Medicine

In hibernators, no specific regulatory pathways or genes activated by OS have yet been identified. The Nrf2-Keap1 and FoxO3a regulatory pathways, common to both hibernators and humans, play a key role in cellular adaptation to stressful conditions, including OS, metabolic changes, and other challenges. In hibernating animals, these mechanisms may be involved in survival in hypoxia, temperature reduction, and other physiological changes during torpor and arousal. Studying these pathways offers potential for developing clinical methods for protecting organs and tissues during hypoxia, ischemia/reperfusion, injury, and other pathologies.

The clinical potential of the Nrf2-Keap1 pathway may include organ protection during ischemia and reperfusion, neuroprotection, and the treatment of toxic injury. Pharmacological activation of the pathway (e.g., with curcumin, resveratrol, and sulforaphane) can protect cells from xenobiotics and electrophilic compounds.

The clinical potential of FoxO3a activation may include: stem cell protection and tissue regeneration, neuroprotection and treatment of neurodegenerative diseases, and control of apoptosis. Modulation of its activity may be useful in the treatment of tumors or inflammatory diseases.

Both pathways are interconnected and can synergistically protect cells from stress.

Promising areas for using hibernators as experimental models:

- Investigation of the mechanisms that allow hibernating animals to activate these pathways in a physiologically controlled manner.
- Development of pharmacological agents that can modulate Nrf2-Keap1 and FoxO3a to protect organs in various pathologies.
- Investigation of the role of these pathways in cellular adaptation to extreme conditions (hypoxia, hypothermia) and their potential application in clinical practice, for example, in tissue or organ cryopreservation.

Thus, understanding the regulatory pathways of Nrf2-Keap1 and FoxO3a in hibernating animals may provide a basis for the development of new therapeutic strategies in medicine.

## 5. Conclusions

The ability of some animals to hibernate is a consequence of adaptations accumulated during evolution at various physiological levels, among which the key is molecular adaptation to hypoxia, which eliminates not only the negative effects of oxygen deficiency on cells, but also the danger of OS upon awakening.

The molecular basis for adaptation to hypoxia in hibernators is the presence of an effective AOS and regulatory mechanisms that ensure extraordinary plasticity of mitochondria, which is especially pronounced when animals emerge from hibernation. During this period, the rate of oxygen consumption in cells increases, and the AOS is activated, including GSH, SOD1/SOD2, CAT, GP, GST and GR. The thioredoxin system and vitamins (A, C, and E) are also involved in the process of protecting tissues from ROS in hibernators. Despite intense interest in the regulation of ROS production in the mitochondria of hibernators, the role of antioxidant systems and their individual components in detoxifying excess ROS products during various periods of natural hibernation remains understudied.

To date, no specific “hibernation genes” have been identified in hibernators responsible for the induction of adaptive reactions during hibernation. However, the mechanisms of regulation of metabolism (metabolic adaptation) present in these animals [3], accumulation of AO, including in excess amounts in individual organs during preparation for hibernation [34], increased production of H2S, a molecule involved in the neutralization of ROS [12,145], as well as the presence of transcription mechanisms [30,106], aimed at reducing the toxic effects of ROS on cells, protect organs from reperfusion and contribute to the successful rehabilitation of animals in the process of arousal from hibernation. Recently, pronounced changes have been found in phosphorylated brain proteins responsible for entering and exiting a state of torpor, including proteins involved in gene expression, DNA maintenance and repair, cellular plasticity, and human diseases. It is noteworthy that the changes were minimal during the transition between active states. The data obtained by these authors provide valuable information about global changes in brain phosphorylation in hibernating mammals, the results of which may be relevant to future therapeutic strategies for brain injury. In addition, according to recent ideas [146], during hibernation, the activity of mitochondria decreases, which, apparently, is important for maintaining cellular circadian rhythms and restoring the redox balance of cells at the level of the entire organism [147].

Thus, the absence of transcriptional “hibernation” specialization and the presence of common mechanisms of regulation of redox balance and cellular circadian rhythms in hibernators and non-hibernators makes it possible to use hibernators as experimental models and can help solve a number of important issues of fundamental biology (strategy of metabolic adaptation to hypothermia) and applied problems in the cardiovascular and traumatic surgery (prevention of ischemic reperfusion syndrome).

## Figures and Tables

**Table 1:** Transcription factors FoxO and NRF2 and the products of their target genes in the regulation of major cellular processes.

Cellular Processes Involving FoxO and NRF2	Target Genes and Their Protein Products					
	FOXO			NRF2		
	Gene	Product/Functions	Reference	Gene	Product/Functions	Reference
**ROS detoxification**	*MnSOD*	Mitochondrial manganese superoxide dismutase/ antioxidant	[32]	*HO-1*	Heme oxygenase-1/ antioxidant	[33]
	*Catalase*	Catalase/ antioxidant	[34,35,36]	*GPX2*	Glutathione peroxidase 2/antioxidant	[37]
	*SESN3*	Sestrin/antioxidant	[38,39]	*GR*	Glutathione reductase/antioxidant	[40]
	*DEPP*	c10orf10/antioxidant	[41]	*GCLC*	The catalytic subunit of the glutamate cysteine ligase/GSH synthesis	[42]
				*GCLM*	Modifying subunit of glutamate cysteine ligase/GSH synthesis	[42,43]
				*EPHX1*	Microsomal epoxide hydrolase1/detoxification	[44]
				*PRDX*	Peroxiredoxin-1/antioxidant enzyme	[45]
				*SRXN1*	Sulfiredoxin-1/antioxidant enzyme	[42]
				*SOD1*	Superoxide dismutase-1/antioxidant enzyme	[46]
				*Nqo1*	NAD(P)H-quinone oxidoreductase 1/antioxidant enzyme	[42,47]
				*UCP1/3*	Uncoupling proteins1/3/mild uncoupling OXPHOS	[48]
	*TXN*	Thioredoxin 1/antioxidant protein	[49]	*TRXR1*	Thioredoxin reductase-1/antioxidant enzyme	[50]
**Participation in transport of xenobiotics, reactive** **metabolites and antioxidant vitamins**				*MRP1*	Multidrug resistance-associated protein 1/transport of xenobiotics, reactive metabolites and participation in the transport of GSH and GSSH out of the cells	[51,52]
				*SCARB1*	Scavenger receptor class B type 1/binding and internalizing modified low density lipoproteins, apoptotic cells and other polyanionic ligands; transport of vitamin E	[53,54,55]
				*SLC7A11*	Cystine/glutamate transporter/maintaining the redox balance between extracellular cystine and cysteine, regulation of GSH synthesis	[56]
				*GST*	Glutathione- S-transferase/xenobiotic detoxification	[57]
				*ALDH3A1*	Aldehyde dehydrogenase 3A1/oxidation of aldehydes into carboxylic acid	[58,59]
				*AKR1C1/3*	Aldoketoreductase family 1 C1/3/involvement in conversion of wide range of substrates, including carbohydrates, steroid hormones, and endogenous prostaglandins	[60,61]
				*AKR7A2*	Aldoketoreductase family 7, A2/involvement in aldehyde metabolism; hepatoprotection	[62,63]
				*CBR1*	Carbonyl reductase-1/ participation in the metabolism of ketones and aldehydes (including drug metabolism)	[64,65]
				*NQO1*	NAD(P)H dehydrogenase (quinone) 1/maintaining redox balance	[66,67]
				*UGT1A1*	Uridine diphosphate glucuronosyl transferase 1 family polypeptide A1/ transformation and elimination of lipophilic molecules	[68]
				*FMO1–FMO5*	Flavin-containing monooxygenases 1–5/detoxification of xenobiotics, biotransformation of lipophilic compounds	[69,70]
	*G6PC*	Glucose-6-phosphatase-α/hydrolysis of glucose-6-phosphate	[71]	*EPHX1*	Epoxide Hydrolase 1/detoxification of low molecular weight compounds	[72]
**Metabolism**	*PEPCK-C*	Phosphoenolpyru-vate carboxykinase C/catalysis of oxalacetate formation, gluconeogenesis, glyceroneogenesis	[73]	*LIPH*	Lipase H/participation in the synthesis of lysophosphatidic acid (LPA); control of cell maturation and proliferation	[74,75]
				*ACOX1*	Acyl-CoA oxidase 1/involvement in the first stage of the peroxisomal β-oxidation of fatty acids	[76]
**Apoptosis**	*BCL2L11*	Bim/proapoptotic protein of the Bcl-2 family, a mediator of apoptosis	[77]	*BID*	Bid/sensor and transducer of apoptotic signals, regulator of the homeostatic-apoptotic switch	[78,79]
	*BNIP3*	BNIP3/proapoptotic protein that mediates apoptosis, necrosis and autophagy; regulator of mitophagy	[80]	*BCL2-like 1*	Bcl-xL/antiapoptotic protein	[81,82]
	*BCL6*	BCL6/transcription repressor; participation in the transcriptional reprogramming	[83,84]	*CASP3*	Caspase-3/responsible for chromatin condensation and DNA fragmentation	[85]
	*TRAIL*	TNF-related apoptosis-inducing ligand/TRAIL selectively induces apoptosis, pro-apoptotic cytokine; TRAIL non-canonical signaling	[86]	*CASP7*	Caspase-7/participation in the final stage of the apoptosis process and formation of apoptotic bodies	[87]
**DNA repair** **and removal of damaged proteins**	*DDB1*	DNA damage-binding protein 1/nucleotide excision repair	[88]	*TP53BP1*	Tumor protein p53 binding protein 1/TP53BP1 recognizes double-chain breaks (DSBs), coordinates DNA repair	[89]
				*RAD51*	RAD51/participation in the repair of double-stranded DNA breaks (DSB)	[90]
				*FKBP5*	FK 506-binding protein 5/regulation of AKT activity, interaction with steroid hormone receptors	[91]
				*SAT1*	Spermidine/spermine N1-acetyltransferase 1/SAT1 promote the expression of DNA damage response pathways	[92]
				*HERPUD1*	Homocysteine -inducible endoplasmic reticulum stress protein with ubiquitin like domain 1/Herpud1 regulate of processes related to endoplasmic reticulum (ER) stress, involved in ER-related protein degradation	[93]
**Biogenesis of mitochondria**				*TFAM*	Mitochondrial transcription factor A/TFAM regulates mitochondrial transcription initiation, participates in the packaging of the mitochondrial genome	[94]
				*NRF1*	Nuclear respiratory factor/NRF1 controls the transcription of genes encoding proteins related to mitochondrial function	[95]
				*Hmox1*	Hemooxygenase-1/HO-1 participates in the regulation of the expression of genes encoding mitochondrial regulatory proteins	[96]
**Autophagy**	*LC3*	LC3/autophagy activator, recruiting substrates	[97]	*Ndp52*	Ndp52/autophagy activator, regulator of transcription	[98,99]
				*p62/SQSTM1*	p62/SQSTM1/ selective autophagy receptor	[100,101]

## Data Availability

No new data were created or analyzed in this study. Data sharing is not applicable to this article.

## Abbreviations

AO	antioxidants
AOS	antioxidant system
AP-1	activating protein-1, transcription factor
ARE	antioxidant response element
Ask1	apoptosis signal-regulating kinase 1
ATP	adenosine triphosphate
BAT	brown adipose tissue
CAT	catalase
CBS	cystathionine β-synthase
CoQ	coenzyme Q
Cul3	ubiquitin ligase
CytC	cytochrome C
Dyrk1	dual-specificity tyrosine-regulated kinase
DNP	2,4-dinitrophenol
ETC	electron transport chain
FAD	flavin adenine dinucleotide
FAO	β-oxidation of fatty acids
FFA	free fatty acids
GP	glutathione peroxidase
GSH	glutathione
GR	glutathione reductase
GST	glutathione-S-transferase
HIF-1	hypoxia-inducible factor 1
HAT	histone acetyltransferase
HDAC1	histone deacetylase 1
HO-1	heme oxygenase-1
H2S	hydrogen sulfide
IL-1β, IL-6	cytokines
iNOS/eNOS	inducible nitric oxide synthase/endothelial NO synthase
Keap1	Kelch-like-ECH-associated protein 1
MafF, MafK, MafG	small Maf family proteins family, transcription factors
MitoQ	mitochondrial antioxidant
MnSOD	mitochondrial superoxide dismutase
NQO1	NAD(P)H:quinone oxidoreductase-1
OAA	oxaloacetate
ONOO^−^	peroxynitrite anion
OS	oxidative stress
OXPHOS	oxidative phosphorylation system
PI3K	phosphoinositide 3-kinase
ROS	reactive oxygen species
SHP protein	small heterodimer partner, orphan nuclear receptor
SQOR	sulfide:quinone oxidoreductase
SOD	superoxide dismutase
Tb	body temperature
TCA	tricarboxylic acid cycle
UCP	uncoupling protein
